# Restoration of ER proteostasis attenuates remote apoptotic cell death after spinal cord injury by reducing autophagosome overload

**DOI:** 10.1038/s41419-022-04830-9

**Published:** 2022-04-20

**Authors:** Elisa Bisicchia, Roberta Mastrantonio, Annalisa Nobili, Claudia Palazzo, Livia La Barbera, Laura Latini, Francesco Millozzi, Valeria Sasso, Daniela Palacios, Marcello D’Amelio, Maria Teresa Viscomi

**Affiliations:** 1 Santa Lucia Foundation, I.R.C.C.S, Rome, Italy; 2 Department of Life Sciences and Public Health Sect. Histology and Embryology, Università Cattolica del S. Cuore, Rome, Italy; 3 Department of Medicine and Surgery, Unit of Molecular Neurosciences, Università Campus Bio-Medico di Roma, Rome, Italy; 4 Division DAHFMO, Unit of Histology and Medical Embryology, Sapienza University of Rome, Rome, Italy; 5 Department of Life Sciences and Public Health Sect. Cell Biology, Università Cattolica del S. Cuore, Rome, Italy; 6 Fondazione Policlinico Universitario “A. Gemelli”, IRCCS, Rome, Italy

**Keywords:** Cell death in the nervous system, Neurodegeneration, Molecular neuroscience

## Abstract

The pathogenic mechanisms that underlie the progression of remote degeneration after spinal cord injury (SCI) are not fully understood. In this study, we examined the relationship between endoplasmic reticulum (ER) stress and macroautophagy, hereafter autophagy, and its contribution to the secondary damage and outcomes that are associated with remote degeneration after SCI. Using a rat model of spinal cord hemisection at the cervical level, we measured ER stress and autophagy markers in the axotomized neurons of the red nucleus (RN). In SCI animals, mRNA and protein levels of markers of ER stress, such as GRP78, CHOP, and GADD34, increased 1 day after the injury, peaking on Day 5. Notably, in SCI animals, the increase of ER stress markers correlated with a blockade in autophagic flux, as evidenced by the increase in microtubule-associated protein 2 light chain 3 (LC3-II) and p62/SQSTM1 (p62) and the decline in LAMP1 and LAMP2 levels. After injury, treatment with guanabenz protected neurons from UPR failure and increased lysosomes biogenesis, unblocking autophagic flux. These effects correlated with greater activation of TFEB and improved neuronal survival and functional recovery—effects that persisted after suspension of the treatment. Collectively, our results demonstrate that in remote secondary damage, impairments in autophagic flux are intertwined with ER stress, an association that contributes to the apoptotic cell death and functional damage that are observed after SCI.

## Introduction

Spinal cord injury (SCI) is a devastating condition that affects millions of persons every year worldwide. Consequently, patients suffer from permanent impairments, for which there are no restorative therapies. In SCI, as in other central nervous system (CNS) pathologies, such as stroke, multiple sclerosis, and traumatic brain injury (TBI), the primary insult entails destructive secondary events that can damage cells that were unaffected or marginally affected by the initial insult. Such secondary damage, which occurs from days to years after the insult, is not limited to the lesion site and can involve remote areas that are functionally related to the primary site of injury, leading to remote cell death [1]. Remote degeneration is a multifactorial phenomenon in which many components become active in specific time frames, and despite its clinical importance in determining outcomes in many CNS pathologies, including SCI and TBI [2, 3], few therapeutic approaches that counteract this subversive mechanism have been proposed.

Following SCI, supra-spinal axotomized neurons enter in a chronic injury state, and the relationship between atrophy and cell death has been largely debated [4, 5]. Axotomized neurons progress through an orderly series of morphological changes, including shrinkage, atrophy, and eventually death as reported in rubro-spinal neurons after cervical axotomy by several groups (for a review: [6]). Nevertheless, despite the controversy of the fate of axotomized neurons, recent studies dealt with the intracellular cascades in rubro-spinal neurons after SCI, reported the occurrence of apoptotic cell death at early time points after injury [7, 8], indicating the importance of the earlier events to correctly tailor neuroprotection.

Autophagy is a controlled intracellular lysosome-mediated degradation pathway that is responsible for recycling and eliminating worn proteins, protein aggregates, and damaged organelles [9, 10]. In particular, cellular homeostasis that is mediated by autophagy relies on the integrity of autophagic flux, the rate of degradation by this pathway [11, 12]. We have previously shown that after SCI, the injury affects the accumulation of dysfunctional autophagosomes, limiting their clearance and, consequently, leading to the build-up of damaged organelles and the ensuing neuronal cell death in remote regions—i.e., in the axotomized neurons of the contralateral red nucleus (RN) [8].

The endoplasmic reticulum (ER) has a significant function in proteostasis, including the folding, maturation, and assembly of proteins that are trafficked along the secretory pathway and the maintenance of cellular calcium homeostasis [13]. Sustained or prolonged ER stress can be cytotoxic, causing apoptotic cell death [14]. To counteract ER stress, cells initiate the unfolded protein response (UPR), an intracellular signaling network that regulates cellular proteostasis and survival [15]. Although ER stress and autophagy involve distinct pathways, they are tightly integrated [16], as supported by studies in various experimental models. For example, persistent ER stress often results in the stimulation of autophagy, likely as a compensatory mechanism to relieve ER stress and ER-associated degradation [17–21], through several canonical UPR pathways [22]. Conversely, impaired UPR or autophagy induces ER stress and the progression of neurodegeneration [23–25]. The link between ER stress and autophagy is supported by accumulating evidence that sustained ER stress damages the integrity of autophagic flux and causes apoptosis in nonalcoholic fatty liver disease [26]. Moreover, it has recently been reported that excessive ER stress/UPR failure disrupts homeostasis in trophoblasts by impairing lysosomal function and consequently inhibiting autophagic flux [27].

Collectively, these studies demonstrate a bidirectional interaction between ER stress and autophagy. The crosstalk between these mechanisms is particularly germane to the pathophysiology of SCI [28–32]. Despite these clear links, whether they are relevant in remote damage after SCI is largely unknown. Determining this aspect is crucial, because impairments in UPR and autophagy in axotomized neurons might promote the engulfment of cellular debris and misfolded proteins by the soma and axons and the loss of their protective functions against cell death after SCI, thus limiting axonal regrowth.

In this study, using a model of spinal cord hemisection, we demonstrate that in remote axotomized neurons of the RN—functionally and anatomically connected to the injured spinal cord—SCI concurrently induces ER stress and reduces lysosomal biogenesis, leading to the blockade of autophagic flux and, consequently, neuronal apoptotic cell death. We also find that pharmacological enhancement of UPR by guanabenz significantly restores homeostasis of the autophagy machinery in remote neurons, consistent with the contribution of ER stress and autophagy dysregulation in mediating remote degeneration after SCI. These effects correlate with increased activation of TFEB—the master regulator of autophagy and lysosome biogenesis—and arrest in neuronal cell death, improved functional recovery that persists over time after injury. Thus, our results indicate that the maintenance of functional UPR is a determinant of proper autophagic flux and, consequently, for remote neuronal survival after SCI.

## Materials and methods

### Animals and spinal cord hemisection

Adult male Wistar rats (200–250 g) were obtained from Harlan (S. Pietro al Natisone, Udine, Italy) and maintained in our animal facilities on a 12:12-h light:dark cycle, receiving food and water ad libitum. All experiments were carried out in accordance with Italian National law in agreement with the ethical guidelines of the European Communities Council Directive (2010/63/EU) for the care and use of laboratory animals and comply with the ARRIVE guidelines. All efforts were made to minimize the number of animals used and their suffering. The animal protocol was approved by the Italian Ministry of Health (Prot. Number: DM30/2014/PR). Special care was taken to use the minimum number of animals required for statistical accuracy. For surgical procedures, the rats were deeply anesthetized by intraperitoneal (i.p.) injections of Rompun (xylazine, 20 mg/ml, 0.5 ml/kg Bayer, Milan, Italy) and Zoletil (tiletamine and zolazepam, 100 mg/ml, 0.5 ml/kg; Virbac, Milan, Italy).

After anesthesia, the skin was treated with betadine and incised, and the layers of muscle were bluntly dissected. A dorsal unilateral laminectomy was performed at C4 to expose the dura-covered spinal cord. The dura was removed using blunt iridectomy scissors and fine forceps. Vertebral segment C4 (corresponding to spinal segment C3/C4) was identified by counting vertebral spines from segment T2. Using sharp iridectomy scissors, a lateral SCI was performed. To ensure the completeness of the injury, a 30-gauge needle was swept through the injury site. Muscle and skin layers were repaired using 5–0 Vicryl sutures and rats were allowed to recover on a heated blanket before being returned to their home cage. Control (CTRL) animals received only a dorsal unilateral laminectomy at C4 to expose the dura-covered spinal cord.

After surgery, animals received an analgesic (2.5 mg per subcutaneous injection of Rimadyl; Pfizer) once per day for 5 days and antibiotics (1.25 mg/250 g body weight intraperitoneal injection of Baytril; Bayer) once per day for 3 days. Animal weights were recorded daily for a week post-surgery, and twice a week thereafter. Bladder function was also assessed for several days post-surgery, but every animal retained bladder function post-operatively. The animals were monitored for hydration and eventual infections until the end of the experiment. SCI were histologically verified postmortem. Incompletely injured or over-hemisected rats were subsequently eliminated from our study.

### Drug treatment

Guanabenz (Sigma #G110) was prepared and dissolved in saline before each injection. Rats received intraperitoneal (i.p.) injections once daily for 5 days (8 mg/kg).

The 8 mg/kg dose was based on previous studies that demonstrated that Guanabenz can enhance UPR in mice [33, 34].

Curcumin analog C1 (1E,4E)-1,5-Bis(2-methoxyphenyl)penta-1,4-dien-3-one (Aktin Chemicals, China), here compound C1, was dissolved in saline and injected once daily for 5 days (10 mg/kg; i.p.). The 10 mg/kg dose is based on previous studies that demonstrated that it is a potent activator of TFEB [35].

Furthermore, for each set of experiments, a group of animals was injected with vehicle (saline; 400 μl i.p., once daily for 5 days).

### Histology and immunohistochemistry

Rats were anesthetized with Rompun (xylazine, 20 mg/ml, 0.5 ml/kg Bayer, Milan, Italy) and Zoletil (tiletamine and zolazepam, 100 mg/ml, 0.5 ml/kg; Virbac, Milan, Italy) and perfused transcardially with 250 ml of saline followed by 250 ml of 4% paraformaldehyde in a phosphate buffer (PB; 0.1 M; pH 0.4) under deep anesthesia. Each brain and spinal cord was removed immediately, post-fixed in the same fixative for 2 h and, after three washes in PB, transferred to 30% sucrose in PB solution at 4 °C until they sinked. Brains and spinal cord were cut into four series of 30-μm-thick transverse sections by means of a freezing microtome and were collected in PB. To assess the extent of the lesion at the spinal cord level as well as the neuronal cell loss in RN following lesion, sections were processed for Nissl-staining [7, 36].

To investigate autophagy activation and mitochondrial dysfunction after ER stress inhibition in damaged rubrospinal neurons, sections were incubated overnight with a cocktail of primary antibodies, including mouse-LC3 (1:200; MBL Int., #M152-3) rabbit anti-LAMP1 (1:500; Cell Signaling; #9091); mouse anti-NeuN (1:200; Millipore, #MAB377); rabbit anti-TFE3 (1:500; Cell Signaling #14779) and rabbit anti-TFEB (1:600; GeneTex #GTX33541). All primary antibody solutions were prepared in PB and 0.3 % Triton X-100 and were incubated 48 h at 4 °C. Each incubation step was followed by three, 5-min rinses in PB. Afterwards, sections were incubated 2 h at RT with a cocktail of secondary antibodies, including Alexa Fluor 647 conjugated donkey anti-mouse (1:200; Invitrogen), Alexa Fluor 543 conjugated donkey anti-rabbit (1:200), and Alexa Fluor 488 conjugated donkey anti-goat (1:200).

For TUNEL assay, brains were cut into 10 μm-thick coronal sections and brain sections were treated according to the manufacturer’s protocol (Click-iT™ Plus TUNEL Assay with Alexa 594 Fluor™ dye; Invitrogen, #C10619). Afterwards, sections were DAPI-counterstained (Termofisher, #62248).

For LAMP1/LC3 immunofluorescent labeling, brains were cut into 20 μm-thick coronal sections using a cryostat and the slices were collected in PB. The sections selected were incubated with digitonin 100ug/ml per 15 min and then in PB + 3% NDS with rabbit anti-LAMP1 and mouse anti-LC3 antibodies overnight. After three washes in PB, sections were incubated with the secondary antibody, including Alexa Fluor 455 conjugated donkey anti-mouse (1:200; Invitrogen), Alexa Fluor 543 conjugated donkey anti-rabbit (1:200). For 3D reconstruction, images were taken as Z-stacks and these Z-stack images were then processed by maximum intensity projection. All samples were acquired with the same laser settings. For quantitative analysis, images were collected from at least 3–4 slices processed simultaneously from and exported for analysis.

Pearson’s correlation coefficient (PCC) was quantified using ZEN V.2.1 software from Zeiss. Quantifications for LC3- and LAMP1-immunoreactive puncta were performed using FIJI software [37].

In general, sections processed for double/triple immunofluorescence were examined under a confocal laser scanning microscope (Zeiss LSM700, Germany) equipped with four laser lines: violet diode emitting at 405 nm (for DAPI), argon emitting at 488 nm, and helium/neon emitting at 543 nm and 633 using a ×40/0.5 NA oil objective. Images were exported in TIFF format, contrast and brightness were adjusted, and final panels were composed using Microsoft PowerPoint.

### Densitometric analysis of fluorescence images

Densitometric analysis of LAMP1 and LC3 proteins was performed on perfused rat brain sections. In order to avoid staining variability among sections and experimental groups, sections of rat brains of the different experimental groups were incubated with the appropriate primary and secondary antibodies at the same time. Furthermore, confocal settings for image capture were maintained constant throughout the acquisition of sections from the different experimental groups.

After background subtraction, LAMP1- or LC3- associated signals were quantified by manually outlining individual RN positive neurons and measuring cell-associated fluorescence intensity with the ImageJ software [38]. The F/A ratio defines mean fluorescence of individual cells (F) normalized to the total cellular surface (A). Quantification was done on 30 cells per animal (*n* = 30 cells/rat; *N* = 5 rats/group).

For TFEB subcellular localization (cytoplasm/nucleus) quantification was performed using five alternate sections of 30-mm regularly spaced throughout the entire RN rostrocaudal extension (*n* = 5 sections/rat; *N* = 5 rats/group). The process was made off-line and only neurons identified by a clear nuclear profile were included for analysis and cells presenting nuclear TFEB expression were expressed as percentage of the total number of RN neurons.

Data collecting for densitometry were done by the experimenter blind to the group analyzed.

### Stereological analyses

To assess the extent of neuronal cell loss in RN following SCI, stereological cell count of RN neurons, was performed on Nissl-stained sections. Quantification of RN neurons was performed using five alternate sections of 30-µm regularly spaced throughout the entire RN rostrocaudal extension. Only neurons with a healthy’ appearance with preserved size, regular nuclear contour, and intense cytoplasmic basophilic substance were included for the analysis. In our study quantitative analyses were limited to the RN of the experimental side projecting to the lesioned spinal cord.

To better appreciate the effects of SCI on RSN cell loss, we related the number of surviving neurons to the number of neurons present in the RN of unlesioned animals (CTRL). Although the lesioned/unlesioned ratio might allow for a direct comparison between sides in the same specimen and decrease the variability, it might underestimate the proportion of surviving neurons. For this reason, to better appreciate the effects of SCI-sal, SCI-Guanabenz, or SCI-CompC1 treatment on RN neuronal loss, we related the number of surviving rubrospinal neurons to the number of neurons present in the RN of unlesioned animals (CTRL). This choice is due to the experimental model employed in the study (spinal cord hemisection) and, considering that the hemisection can minimally affect the “intact” side of the spinal cord causing a neuronal loss in the RN projecting to the unlesioned side, we related the number of surviving neurons to the number of neurons present in the RN of unlesioned animals (CTRL) [7].

Using the Stereo Investigator System (MicroBrightField Europe e.K., Magdeburg, Germany), an optical fractionator, the stereological design was applied to obtain unbiased estimates of total Nissl-stained neurons. A stack of MAC 5000 controller modules (Ludl Electronic Products, Ltd. Hawthorne, NY, USA) was configured to interface an Olympus BX 50 microscope with a motorized stage and a HV-C20 Hitachi color digital camera with a Pentium II PC workstation. A three-dimensional optical fractionator counting probe (*x*, *y*, *z* dimensions of 50 × 50 × 10 μm, respectively) was applied. The RN was outlined using the ×5 objective, while the ×100 oil immersion objective was used for marking the neuronal cells. The total number of rubrospinal neurons was estimated according to the formula given below:

$$N = {\rm{SQ}} \times 1/{\rm{ssf}} \times 1/{\rm{asf}} \times 1/{\rm{tsf}}$$

where SQ represents the total number of neurons counted in all optically sampled fields of the RN, ssf is the section sampling fraction, asf is the area sampling fraction, and tsf is the thickness sampling fraction.

### Tissue extraction and immunoblotting

The brains were immediately dissected in fresh PBS and the cerebellum was separated from the entire brain and discharged and afterwards the midbrain was dissected by using a rat brain matrix with 1 mm coronal sections slice intervals. Afterwards the sections obtained at the level of the ventral tegmental area, of the substantia nigra and aqueduct were collected (2/3 for each rat) and, for each section, the RN projecting to the lesioned side of the spinal cord was isolated under a dissection microscope using the above-mentioned brain structures as landmarks. Once isolated, the RNs were homogenized in lysis buffer (320 mM sucrose, 10% glycerol, 50 mM NaCl, 50 mM TRIS-HCl, pH. 5, 1% Triton X-100, 1 mM PMSF) with protease inhibitor cocktail (Sigma, P8340), incubated on ice for 30 min and centrifuged at 13,000 × *g* for 20 min. The total protein content of the resulting supernatant was determined. Proteins were applied to SDS-PAGE and electroblotted on a PVDF membrane. Immunoblotting analysis was performed using chemiluminescence detection kit.

To assess the release of cyt-c in the cytosol we separated mitochondrial and cytosolic fraction. Briefly, RNs were homogenized in Buffer A (320 mM sucrose, 1 mM EDTA, 50 mM TRIS-HCl, pH 7.4, 1 mM DTT, and 1 mM PMSF), with protease inhibitor cocktail (Sigma, P8340) by 30 strokes with a glass Pyrex micro homogenizer (Sigma). The homogenate was centrifuged at 1000 × *g* for 10 min, and the resulting supernatant was centrifuged at 10,000 × *g* for 20 min to obtain the mitochondrial pellet and the supernatant. The mitochondria-containing pellet was washed three times with buffer B (250 mM sucrose, 1 mM EGTA–Sigma, E3889, 10 mM TRIS-HCl pH. 4) by centrifugation for 10 min at 10,000 × *g*. The supernatant was centrifuged at 100,000 × *g* for 1 h to generate the cytosolic fraction [7].

As TFEB is known to be present in both the nucleus and the cytosol, we separated cytosolic and nuclear fractions. Briefly, the tissues were pestle homogenized in buffer A and centrifuged for 5 min at 3000 rpm in cold. The supernatants were used as cytoplasmic extracts. For nuclear fractions, the pellet was dissolved in buffer C (20 mM HEPES, pH 7.9, 400 mM NaCl, 1 mM EDTA, and 1 mM dithiothreitol plus protease inhibitor cocktail) and vortexed vigorously for 15 min in the cold. The suspension was incubated for 30 min at 4 °C under constant shaking. The samples were spun at 14,000 rpm for 10 min at 4 °C. The supernatants were diluted with buffer D (20 mM HEPES, pH 7.9, and 1 mM EDTA plus protease inhibitor cocktail) at five final volumes and used as nuclear fractions. The protein signals were detected using Super Signal West Pico Chemiluminescent substrate (Pierce Biotechnology Inc., USA) and normalized to actin (cytosol) or H3 (nuclear) levels used as loading control.

The relative levels of immunoreactivity were determined by densitometry using ImageJ software (NIH; USA). Samples were incubated overnight with the following primary antibodies: mouse anti-cytochrome-c (1:1000; BD Pharmingen, UK); rabbit polyclonal anti-p62 (1:1000; MBL International; #PM045); mouse monoclonal anti-LC3 (1:250; NanoTools #0231-100/LC3-5F10); goat anti-Grp78 (1:500; Santa Cruz Biotechnology; #sc1051); rabbit anti-GADD34 (1:500; Santa Cruz Biotechnology; #sc824); mouse anti-CHOP (1:500; Santa Cruz Biotechnology; #sc7351); rabbit anti-eIF2α (1:1000 Cell Signaling #D968); rabbit anti-p-eIF2α (1:1000 Cell Signaling; #3597); rabbit anti-Bcl-2 (1:500; Santa Cruz Biotechnologies, #492); mouse anti-Bax (1:2000; Santa Cruz Biotechnologies; #sc7480); and mouse anti-GAPDH (1:5000; Abcam; #ab8245); rabbit anti-LAMP1 (1:100; Cell Signaling; #9091); mouse anti-LAMP2 (1:500; Santa Cruz Biotechnologies; #sc19991); rabbit anti-TFE3 (1:500; Cell Signaling #14779) and rabbit anti-TFEB (1:600; GeneTex #GTX33541). goat anti-cathepsin-D (CTSD 1:1000; Santa Cruz Biotechnologies; #sc6487); rabbit anti-cleaved caspase-3 (1:1000; Cell Signaling; #9664); rabbit anti-Histone H3 (1:2000; Abcam; #ab1791). Membranes were then incubated with the appropriate horseradish peroxidase-conjugated secondary antibodies. Immunoreactive bands were detected by using an enhanced chemiluminescence kit (ECL; Amersham Biosciences).

### Quantitative real-time RT-PCR

RNA was extracted from RN using Total RNA Purification Kit from Norgen Biotek (Canada) according to the manufacturer’s instructions. Then, 1 µg of total RNA was used for RT reaction by using the SuperScript VILOTM cDNA Synthesis Kit (Invitrogen). The following RT-PCR program was used: 25 °C for 10 min, 42 °C for 60 min, and 85 °C for 5 min. The expression of the different primers was assessed by quantitative RT-PCR(qRT-PCR) using Syber Green PCR Master Mix (Applied Biosystem) as a fluorescent dye to monitor cDNA amplification. The following PCR program was used: 95 °C for 10 min, 40 cycles at 95 °C for 15 s, and 60 °C for 60 s. The primers used were listed in Table 1.

**Table 1:** Rat primers used for the quantitative RT-PCR.

Primers	Forward	Reverse
GADD34	TACCTGGACAGAAAGCCAGCA	AGAAGTGCACCTTTCTACCCT
GRP78	ACGAAGGTGAACGACCCC	GCAGGAGGGATTCCAGTCAG
CHOP	GGAGCTGGAAGCCTGGTATG	GCTAGGGATGCAGGGTCAAG
LAMP1	GGGGAACAAGAGCAGAGTCC	GTGCTGAACGTGGGCTCTAT
LAMP2	GTCGTCACTTGTCCTGAGGG	TCAATGCATCGGACCGAACT
p62/SQSTM1	GCTGCCCTGTACCCACATCT	CGCCTTCATCCGAGAAAC
HEXA	GCCCCAGTACATCCAAACCT	TACGGTAGCGTCGAAAGGC
ATP6V1H	TCCAGGACCTTAGAATCTTGACA	CTCAATAACCCGTTTGCCCC
CTSD	AACAATGTGCTCCCGGTCTT	GTGCCGCCAAGCATTAGTTC
GAPDH	GGACCAGGTTGTCTCCTGTG	CATTGAGAGCAATGCCAGCC

GAPDH was used as a housekeeping gene for quantity normalization. Then, 1 µl of the first strand of cDNA product was used for amplification (in triplicate) in a 20 µl reaction solution, containing 10 µl of Syber Green PCR Master Mix (Applied Biosystem) and 1 mmol of each primer. Fold change was determined by using the ΔΔC(t) method.

### Beam-walking test

Animals were examined with a fine-motor test paradigm (beam-walking test) at 0 and 1, 3, and 5 days after surgery. In the test, the locomotion of the rats was evaluated pre-operatively and post-operatively using a beam-walking task with an elevated narrow beam (150 cm long × 2.5 cm wide). The worst score (‘0’) was given if the rat was unable to traverse the beam and could neither place the affected limbs on the horizontal surface nor maintain balance. A score of ‘1’ was given if the rat was unable to traverse the beam or to place the affected limbs on the horizontal surface of the beam but was able to maintain balance. A score of ‘2’ was given if the rat was unable to traverse the beam but placed the affected limbs on the horizontal surface of the beam and maintained balance. A score of ‘3’ was given if the rat used the affected limbs in less than half of its steps along the beam. A score of ‘4’ was given if the rat traversed the beam and used the affected limbs to aid with more than 50% of its steps along the beam. A score of ‘5’ was given if the rat traversed the beam and used the affected limbs to aid with less than 50% of its steps along the beam. A score of ‘6’ was given if the rat traversed the beam normally with no more than two-foot slips. The week before surgery, animals were trained to run the narrow beam for a food reward once daily, 5 days per week. All the animals in all groups underwent the motor behavior test to ensure that their performance score before surgery (day 0) was 6. An investigator who was blinded to the experimental groups conducted these experiments.

### Experimental design and statistical analysis

The number of animals used in each experiment is listed in the figure legend section. The numbers of animals used for biochemical analysis (Wb and qRT-PCR), locomotor test, and morphological analysis were based on our previous experience with the techniques and on the basis of a sample size calculation performing a power analysis (G Power 3.1 software). In all cases, we assumed a probability equal to 0.05 and a test power equal to 95%, while Δ and standard deviations were based on previous experiments from our group [8]. For statistical analyses, *t*-test or one-way, two-way (multiple groups) or repeated-measure analysis of variance (ANOVA) followed, in cases of significance, by a Bonferroni post hoc test was applied. See figure legends for more details. Values of *p* ≤ 0.05 were considered to be statistically significant. In the box-and-whisker plots, the center line denotes the median value, edges are upper and lower quartiles, whiskers show minimum and maximum values and points are individual experiments. Statistical analyses were carried out by GraphPad Prism 6.0 (GraphPad software for Science, San Diego, CA). All quantitative analyses were conducted blind to the animal’s experimental groups.

## Results

### SCI induces ER stress and disrupts autophagic flux in remote neurons

**Fig. 1:**
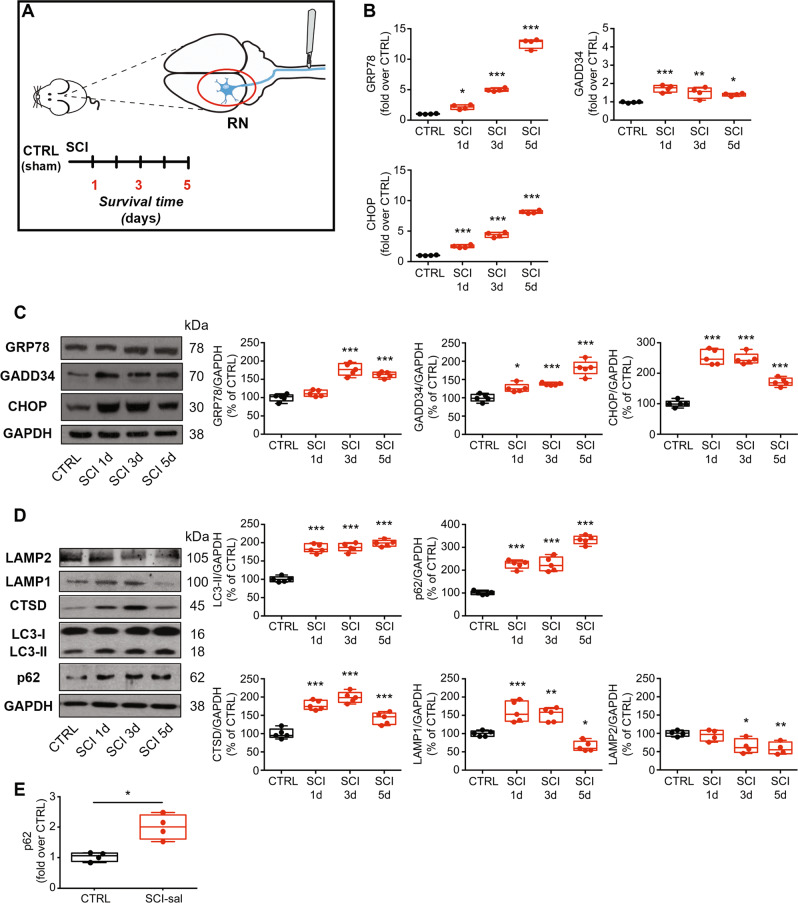
Spinal cord injury induces ER stress and autophagy flux disruption in remote regions. **A** Schematic of the protocol used in the study. Adult rats underwent spinal cord injury (SCI), or sham lesion (CTRL). At day 1, 3 and 5 SCI animals were sacrificed and the red nucleus (RN) contralateral to the lesion side was extracted and processed for biochemical analyses or analyzed on fixed-brain sections. **B** Box-and-whisker plots of GRP78, GADD34 and CHOP mRNA level in control animals (CTRL) and after spinal cord injury (SCI) at various time points after damage (1, 3, and 5 days), expressed as fold over CTRL (*N* = 5 rats per group; one-way ANOVA, *p* = 511.1 GRP78; *p* = 11.87 GADD34; *p* = 532.3 CHOP) ***p < 0.001, **p < 0.01, *p < 0.05 vs CTRL. **C** Representative western blot and densitometric box-and-whisker plots from the CTRL and SCI animals at different time points after injury showing the levels (expressed as % of CTRL) of GRP78, GADD34 and CHOP normalized to GAPDH used as loading control (*N* = 5 rats per group; one-way ANOVA, *p* < 0.0001 GRP78; *p* < 0.0001 GADD34; *p* = 0.0001 CHOP) ***p < 0.001, **p < 0.01, *p < 0.05 vs CTRL. **D** Representative western blot and densitometric box-and-whisker plots from the different groups showing the levels (expressed as % of CTRL) of LC3, p62, CTSD, LAMP1, and LAMP2 normalized to GAPDH used as loading control (*N* = 5 rats per group; one-way ANOVA, *p* < 0.0001 LC3-II; *p* < 0.0001 p62; *p* < 0.0001 CTSD; *p* < 0.0001 LAMP1; *p* < 0.01 LAMP2). ***p < 0.001, **p < 0.01, *p < 0.05 vs CTRL. In all box-and-whisker plots of the present study the centre line shows the median value, edges are upper and lower quartiles, whiskers show minimum and maximum values, and each point is an individual animal. **E** Box-and-whisker plots of p62/SQSTM1 mRNA level in CTRL and SCI-sal as fold over CTRL (*N* = 4 rats per group; Unpaired *t*-test with Welch's correction *p* = 0.0125) *p < 0.05.

To examine the interplay between ER stress and autophagy in remote regions after SCI, we analyzed their kinetics. Because the PERK branch of the UPR is crucial in SCI [25, 39], we considered the mRNA and the protein levels of several key markers of this arm—GRP78, GADD34, and CHOP—in the RN of control (CTRL) and lesioned animals (SCI) at 1, 3, and 5 days after injury (Fig. 1A). The mRNA levels of all ER markers increased in SCI animals, starting from 1 day after the injury, and continued to rise or remained elevated versus CTRL (Fig. 1B). In the protein analysis, we noted a significant increase in GRP78, GADD34, and CHOP in SCI animals compared to CTRL (Fig. 1C).

Further, autophagic activity in remote regions was monitored, based on changes in the autophagosomal protein LC3-II, the autophagy substrate p62/SQSTM1, and the lysosomal markers LAMP1, LAMP2, and cathepsin D (CTSD). Consistent with previous findings on the activation of autophagy after SCI ([8]), we noted a time-dependent increase in the levels of LC3-II (Fig. 1D) that paralleled the upregulation of p62/SQSTM1 at the protein level (Fig. 1D). Considering that in response to various stresses p62 accumulation is also driven by pre-translational regulation, we measured its mRNA level in CTRL and after SCI (SCI 5 days). The results showed that p62 mRNA level increased dramatically in SCI animals compared to CTRL (two fold over CTRL; *p* < 0.01) (Fig. 1E). These results showed that both p62 mRNA and protein levels increased dramatically in SCI animals compared to CTRL.

Moreover, we examined lysosomal function by measuring CTSD, LAMP1, and LAMP2 levels. As shown in Fig. 1D, CTSD rose significantly in SCI animals compared to CTRL 1 and 3 days after injury and, although slightly decreased at day 5, it remained significantly elevated up to 5 days. Conversely, at the different time points analyzed, LAMP1 rose significantly in SCI animals compared with CTRL 1 and 3 days after injury, while on day 5 it fell down, reaching a level slightly lower than CTRL (Fig. 1D). LAMP2 level remained quite similar to CTRL at 1 day after injury, while on day 3, and more on day 5, it fell down, reaching a level significantly lower than CTRL (Fig. 1D), suggesting that excessive ER stress induced by SCI downregulated lysosomal proteins and, consequently, impairs autophagic flux.

Collectively, our findings show that after SCI, in remote regions, the kinetics of ER stress and autophagy parallel each other, implicating a link between ER stress and the disruption of autophagic flux.

### Pharmacological enhancement of UPR restores autophagic flux after SCI

**Fig. 2:**
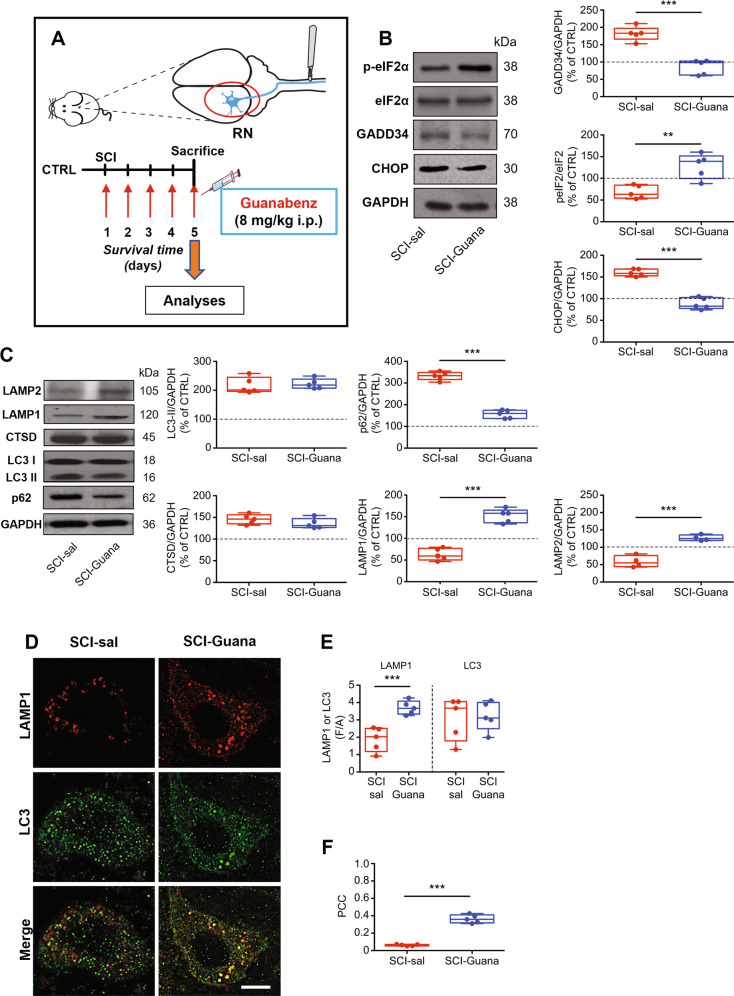
Enhancement of UPR by guanabenz reduces the SCI-induced effects on ER stress and autophagy flux. **A** Schematic of the protocol used in the study. Adult rats underwent spinal cord injury (SCI) received guanabenz (8 mg/Kg i.p. once a day) or saline for 5 days. At day 5 animals were sacrificed and the red nucleus (RN) contralateral to the lesion side was extracted and processed for biochemical analyses or analyzed on fixed-brain sections. **B** Representative western blot and densitometric box-and-whisker plots from the SCI-saline (SCI-sal) and SCI-Guanabenz (SCI-Guana) treated animals showing the levels (expressed as % of CTRL) of p-eIF2α/eIF2α, GADD34 and CHOPp normalized to GAPDH used as loading control (*N* = 5 rats per group; Unpaired *t* test with Welch’s correction *p* = 0.0057 p-eIF2α/eIF2α; *p* < 0.0001 GADD34; *p* < 0.0001 CHOP) ***p < 0.001, **p < 0.01. **C** Representative western blot and densitometric box-and-whisker plots from the SCI-sal and SCI-Guana groups showing the levels (expressed as % of CTRL) of LC3, p62, CTSD, LAMP1, and LAMP2 normalized to GAPDH used as loading control (*N* = 5 rats per group; Unpaired *t* test with Welch’s correction *p* = 0.7270 LC3-II; *p* = 0.0001 p62; *p* = 0.0001 CTSD; *p* = 0.0001 LAMP1; *p* < 0.001LAMP2). ***p < 0.001, **p < 0.01. **D** Confocal Z-stack double immunolabelling for LAMP1 (red) and LC3 (green) puncta in RN neurons from SCI-sal and SCI-Guana animals (scale bar = 10 μm). Single red (LAMP1) or green (LC3) puncta indicates single lysosomes or autophagosomes, respectively. Yellow puncta (merge of red and green) indicates lysosomes fused with autophagosomes (autophagolysosomes). **E** Box and whisker plots showing the densitometric analyses of LAMP1 and LC3 immunostaining in neurons of RN (*N* = 5 rats for group; 30 cells/animal; Unpaired *t* test with Welch’s correction *p* = 0.0052 LAMP1; *p* = 0.5029 LC3) ***p < 0.001. **F** Box and whisker plots showing the co-localization between LAM*P*1 and LC3 immunostaining in the RN neurons from SCI-sal and SCI-Guana groups. LAMP1-LC3 co-localization is expressed as Pearson’s coefficient of correlation (PCC) (*N* = 5 rats for group; 30 cells/animal; Unpaired *t* test with Welch’s correction *p* < 0.0001) ***p < 0.001.

To determine whether the prolonged ER stress observed influences autophagic flux, we treated SCI rats with guanabenz (Fig. 2A), an FDA-approved, centrally acting oral antihypertensive drug that enhances the UPR by keeping prolonged eIF2α phosphorylation [33, 40].

Five days after daily treatment with guanabenz, we first assessed its efficacy on interfering with ER stress-signaling, such as eIF2α phosphorylation, GADD34, and CHOP levels.

As shown in Fig. 2B, after SCI, guanabenz (SCI-Guana) significantly decreased GADD34 and restored eIF2α phosphorylation over CTRL levels (Fig. 2B). Furthermore, CHOP levels fell significantly in the SCI-Guana versus SCI-sal group, demonstrating that guanabenz has broad effects on ER stress signaling (Fig. 2B). Moreover, in our analysis of autophagy, after SCI, guanabenz did not significantly affect the LC3-II or CTSD levels (Fig. 2C). LC3-II and CSTD levels were elevated in the RN after SCI, and they did not rise further on guanabenz treatment (SCI-sal vs SCI-Guana; Fig. 2C). Conversely, guanabenz significantly altered the levels of p62/SQSTM1, LAMP1, and LAMP2. Specifically, in SCI-Guana animals, p62/SQSTM1 was downregulated compared to the SCI-sal group (Fig. 2C), whereas LAMP1 and LAMP2 levels were upregulated (Fig. 2C).

To confirm these findings, we analyzed the colocalization of LC3 and LAMP1 by confocal analysis to assess autophagosome-lysosome fusion. By immunostaining, LAMP1 expression was significantly higher in the SCI-Guana versus SCI-sal group (Fig. 2D–E), whereas LC3 levels were similar between groups (Fig. 2D–E). Notably, the Pearson’s coefficient of correlation (PCC) for colocalization was higher in the SCI-Guana versus SCI-Sal group (PCC_SCI-Guana_ = 0.328 vs PCC_SCI-sal_ = 0.054; Fig. 2F), suggesting that guanabenz increased the number of autophagolysosomes compared to SCI-sal.

These results demonstrate that ER stress and autophagic flux are closely linked and that the modulation of the ER stress response by guanabenz increases lysosome biogenesis and restores the autophagic flux altered after SCI.

### Pharmacological enhancement of UPR mitigates remote apoptotic cell death and improves functional recovery after SCI

**Fig. 3:**
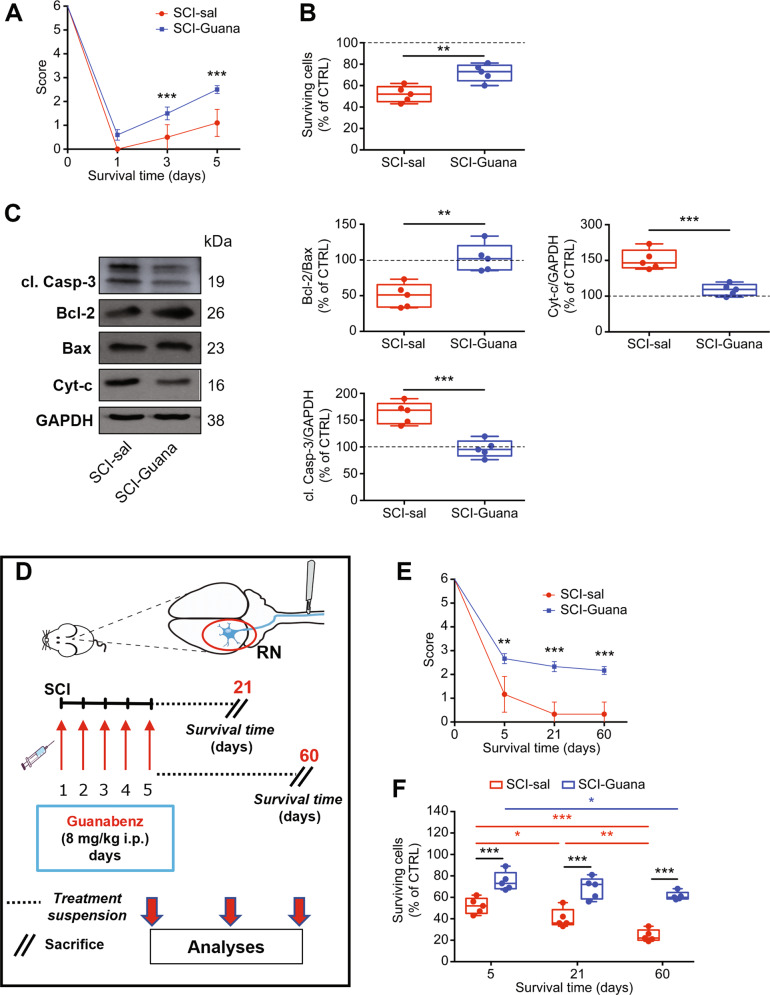
Pharmacological treatment with guanabenz reduces neuronal death induced by SCI and improves functional recovery. **A** Time course of functional recovery measured by Beam-walking test showing the score of SCI-sal and SCI-Guana rats (*N* = 17 mice/group, m/f = 10/7; Two-way repeated measures ANOVA, (time × treatment: time *p* < 0.0001; treatment *p* = 0.0002; interaction: time × treatment *p* < 0.0001) **p < 0.001; ***p < 0.0001. **B** Box-and-whisker plots showing the percentage of surviving neurons in RN of SCI-sal and SCI-Guana rats measured by stereological analysis (*N* = 5 rats/group, m/f = 3/2; Unpaired *t* test with Welch’s correction *p* = 0.0036) **p < 0.001. **C** Representative immunoblots and densitometric box-and-whisker plots showing the level of cytochrome-c (cyt-c) released in the cytoplasm, cleaved Caspase-3 (cl. Casp-3), Bcl-2/Bax ratio normalized to GAPDH used as loading control in the SCI-sal and SCI-Guana groups (*N* = 5 rats/group, m/f = 3/2; Unpaired *t* test with Welch’s correction *p* = 0.0049 Bcl-2/bax ratio; *p* = 0.0019 cyt-c; *p* = 0.0005 Caspase-3) **p < 0.001; ***p < 0.0001. **D** The experimental protocol used in this section to investigate the long-lasting effects of guanabenz treatment. Adult rats underwent spinal cord injury (SCI) received guanabenz (8 mg/Kg i.p. once a day) or saline for 5 days. After that, the treatment was suspended and animals were divided into two groups: a group of animals was left to survive another 16 days after the end of treatment and sacrificed at day 21; the second group was left to survive another 55 days after the end of treatment and sacrificed 60 days after injury. **E** Time course of functional recovery measured by Beam-walking test showing the score of SCI-sal and SCI-Guana rats (*N* = 6 rats/group, m/f = 4/2; Two-way RM ANOVA (time × treatment: time *p* < 0.0001; treatment *p* = 0.0001; interaction: time × treatment *p* < 0.0001) **p < 0.001; ***p < 0.0001. **F** Box-and-whisker plots showing the percentage of surviving neurons in RN of SCI-sal and SCI-Guana rats at different time points (5, 21, and 60 days after injury) measured by stereological analysis (*N* = 5 mice/group, m/f = 3/2; Unpaired *t* test with Welch’s correction *p* = 0.0011) **p < 0.001.

Based on the ability of guanabenz to enhance the UPR and restore autophagic flux, we determined its modulatory effects on functional recovery and remote neuronal survival. We first examined whether guanabenz alters the functional-behavioral outcomes that are observed after SCI. Saline-treated (SCI-sal) and SCI-guanabenz-treated (SCI-Guana) animals were evaluated for motor performance using the beam walking test at baseline (before damage, survival time 0) and 1, 3, and 5 days after injury. Notably, after SCI, treatment with guanabenz significantly accelerated functional recovery, based on beam walking test scores (Fig. 3A). Starting at 3 days, the SCI-Guana group had better scores than the SCI-sal group, a difference that was more pronounced 5 days after injury (Fig. 3A).

Because neurological recovery following brain injury or SCI is highly influenced by neuronal survival in key brain regions, we reasoned that the neurological improvement that was observed in SCI-Guana animals—i.e., higher beam walking test scores—would be accompanied by greater neuronal survival. By using a quantitative stereological analysis of Nissl-stained neurons, we showed that SCI induced a significant neuronal loss in RN of SCI-sal group (Fig. 3B), further confirmed by TUNEL assay (Supplementary Fig. 1). Furthermore, the percentage of surviving neurons was significantly higher in SCI-Guana versus SCI-sal animals, indicating that guanabenz treatment following SCI significantly mitigates the rate of neuronal degeneration induced by SCI (Fig. 3B). This result prompted us to further determine the modulatory effects of guanabenz on the SCI-induced apoptotic pathway that has been showed effective in inducing neuronal death after SCI [7]. Notably, guanabenz-dependent enhancement of UPR was associated with a significant increase in the Bcl-2/Bax ratio (Fig. 3C) and a reduction in cytochrome-c release from damaged mitochondria (Fig. 3C) and in cleaved caspase-3 (Fig. 3C). Collectively, these findings suggest that guanabenz-dependent enhancement of UPR significantly improves functional recovery in injured animals and halted apoptotic remote cell death due to SCI.

Next, we examined whether guanabenz treatment modulates or maintains functional outcomes and neuronal survival at later time points (21 days and 60 days after injury) (Fig. 3D). To this end, after SCI, we treated animals with guanabenz or saline for 5 days and then suspended the treatment for 16 (SCI-21 Guana or SCI-21 sal) and 55 days (SCI-60 Guana or SCI-60 sal) (Fig. 3D), and their motor performance was evaluated using the beam walking test. Interruption of treatment did not induce long-term motor alterations (Fig. 3E). Whereas scores were slightly worse in SCI-sal animals at 21 and 60 days after injury than at 5 days, such scores were nearly comparable in SCI-Guana animals (Fig. 3E). Notably, the SCI-Guana groups had better scores than SCI-sal animals at 21 and 60 days after injury (Fig. 3E).

Similarly, our stereological analysis revealed that at both time points after injury, the percentage of surviving neurons in the SCI-Guana groups was significantly higher than in the SCI-sal groups (Fig. 3F) but similar compared with at 5 days after guanabenz treatment (Fig. 3F). Conversely, in the SCI-sal group, at 21 and 60 days after injury, this percentage was significantly lower than at 5 days (Fig. 3F), suggesting a faster decline in neuronal survival in SCI-sal versus SCI-Guana animals.

These results suggest that the enhancement of UPR and the restoration of autophagic flux at early time points are crucial in improving and maintaining functional recovery and slowing remote neuronal degeneration induced by SCI.

### Guanabenz restores autophagic flux by modulating TFEB

Once determining the long-term effects of guanabenz, we wondered whether the ER stress-induced lysosomal dysfunction that is observed 5 days after injury is due to impaired activity of the MiT/TFE family members, including MITF, TFEB, TFE3, and TFEC, which play crucial roles in the regulation of lysosomal function and autophagy [41, 42]. Given prior evidence that identifies both TFE3 and TFEB as primary factors in the link between ER stress and autophagy [25, 43], we investigated their levels in the different experimental conditions, namely in CTRL, SCI-sal, and SCI-Guana groups.

**Fig. 4:**
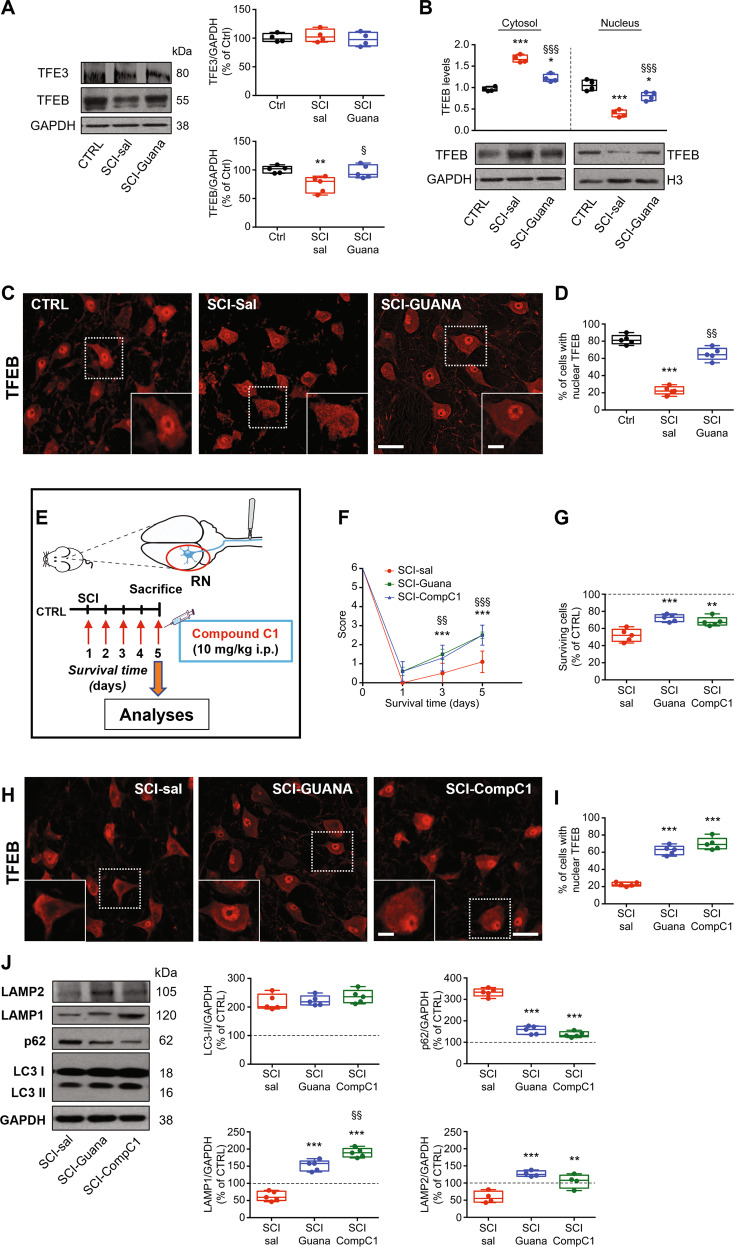
Enhancement of UPR by guanabenz after SCI restores autophagy flux by modulating TFEB. **A** Representative immunoblots and densitometric box-and-whisker plots showing the total TFEB and TFE3 levels in CTRL, SCI-sal and SCI-Guana groups (*N* = 4 rats/group, m/f = 2/2; one-way ANOVA *p* < 0.0001 TFEB; one-way ANOVA *p* = 0.1368); **p < 0.01 vs CTRL; ^§^*p* < 0.05 vs SCI-sal. **B** Representative immunoblots and densitometric box-and-whisker plots showing the TFEB cytosolic level (normalized to GAPDH) and nuclear level (normalized to H3) in CTRL, SCI-sal and SCI-Guana groups (*N* = 4 rats/group, m/f = 2/2; one-way ANOVA *p* = 0.0001); ***p < 0.001 vs CTRL; *p < 0.05 vs CTRL; ^§§§^*p* < 0.001 vs SCI-sal. **C** Representative confocal images of TFEB immunofluorescence showing the subcellular compartimentalization of TFEB immunostaining in RN neurons of CTRL, SCI-sal and in SCI-Guana groups (scale bar = 20 μm; inset = 5 μm). **D** Box and whisker plots showing the percentage of neurons of RN with nuclear expression of TFEB in CTRL, SCI-sal and SCI-Guana (*n* = 5 sections/rat; *N* = 5 rats/group; m/f = 3/2; one-way ANOVA *p* < 0.0001). The process was made off-line and only neurons identified by a clear nuclear profile were included for analysis. Cells presenting nuclear TFEB expression were expressed as percentage of the total number of RN neurons. ***p < 0.001 vs CTRL; ^§§^*p* < 0.001 vs SCI-sal. **E** The experimental protocol used for assessing the role of TFEB in restoring the SCI-induced effects. Adult rats underwent spinal cord injury (SCI) received compound C1 (10 mg/Kg i.p. once a day) or saline for 5 days. At day 5 animals were sacrificed and the red nucleus (RN) contralateral to the lesion side was extracted and processed for biochemical analyses or analyzed on fixed-brain sections. **F** Time course of functional recovery measured by Beam-walking test showing the score of SCI-sal, SCI-Guana and SCI-CompC1 rats (*N* = 6 mice/group, m/f = 4/2; Two-way RM ANOVA, (time × treatment: time *p* < 0.0001; treatment *p* = 0.0002; Interaction: time × treatment *p* < 0.0001) ***p < 0.001 SCI-sal vs SCI-Guana; ^§§^*p* < 0.001 SCI-sal vs SCI-CompC1; ^§§§^*p* < 0.001 SCI-sal vs SCI-CompC1. **G** Box-and-whisker plots showing the percentage of surviving neurons in RN of SCI-sal, SCI-Guana and SCI-CompC1 rats measured by stereological analysis (*N* = 5 rats/group, m/f = 3/2; one-way ANOVA *p* = 0.0003) ***p < 0.0001, **p < 0.001 vs SCI-sal. **H** Representative confocal images TFEB immunofluorescence from RN showing the compartimentalization of TFEB immunostaining in neurons of SCI-sal, SCI-Guana and SCI-CompC1 groups (scale bar = 20 μm; inset = 5 μm). **I** Box and whisker plots showing the percentage of neurons of RN with nuclear expression of TFEB in SCI-sal, SCI-Guana, and SCI-CompC1 (*n* = 5 sections/rat; *N* = 5 rats/group; m/f = 3/2; one-way ANOVA *p* < 0.0001) ***p < 0.001 vs SCI-sal. **J** Representative western blot and densitometric box-and-whisker plots from the SCI-sal, SCI-Guana and SCI-CompC1 groups showing the levels (expressed as % of CTRL) of LC3, p62, LAMP1, and LAMP2 normalized to GAPDH used as loading control (*N* = 5 rats per group; m/f = 3/2; one-way ANOVA *p* = 0.4094 LC3-II; *p* < 0.0001 p62; *p* < 0.0001 LAMP1; *p* < 0.001 LAMP2) ***p < 0.0001 vs SCI-sal. ^§§^*p* < 0.01 vs SCI-Guana.

As shown in Fig. 4A, SCI markedly decreased total levels of TFEB but did not affect the TFE3 levels. After guanabenz treatment, total levels of TFEB were significantly higher than in SCI-sal but lower, although not significant, versus CTRL (Fig. 4A), while total levels of TFE3 were not affected (Fig. 4A). The TFE3 results were confirmed by immunostaining, which showed that TFE3 subcellular expression in the neurons of RN was quite similar in the different experimental conditions considered (Supplementary Fig. 2A), as well as the percentage of RN neurons with nuclear TFE3 expression (Supplementary Fig. 2B).

Considering the TFEB changes observed, in order to clarify the function of TFEB after SCI, we first checked its subcellular localization by Western blot. TFEB is localized in the cytoplasm under normal conditions. However, in response to certain stimuli, such as starvation or injury, TFEB translocates to the nucleus and activates a transcriptional program [44, 45]. As shown in Fig. 4B, SCI markedly increased cytosolic levels of TFEB, and decreased the nuclear levels compared with CTRL (Fig. 4B). After guanabenz treatment, cytosolic levels decreased and nuclear levels were significantly higher than in SCI-sal but lower versus CTRL (Fig. 4B). These results were confirmed by TFEB immunostaining, which showed that TFEB was widely expressed in the cytosol and nuclei of RN neurons in the CTRL group (Fig. 4C) but mainly in the cytosol of neurons and nearly absent in neuronal nuclei after SCI (SCI-sal; Fig. 4C). After guanabenz treatment (SCI-Guana), TFEB was confined primarily to the nuclear compartment (Fig. 4C). Our quantitative analysis of TFEB subcellular localization confirmed that the percentage of neurons with nuclear TFEB expression was lower in the SCI-sal group than in CTRL (Fig. 4D) but significantly higher in SCI-Guana versus SCI-Sal (Fig. 4D), although lower than in CTRL, implicating TFEB in ER stress-mediated dysfunction of autophagy after SCI and guanabenz treatment was effective in modulating TFEB.

To ascertain that guanabenz treatment was directly acting on TFEB, we performed qRT-PCR analysis for the mRNA levels of some TFEB target genes, such as Lamp1, Lamp2, Hexa and ATP6V1A. We found that the mRNA levels of these TFEB target genes were significantly higher in SCI-Guana group compared to SCI-sal (Supplementary Fig. 1C). Collectively, these data indicate that guanabenz was effective in improving TFEB-mediated lysosomal biogenesis.

To validate TFEB results, we treated SCI animals with compound C1 for 5 days (Fig. 4E) and compared its effects to those of saline and guanabenz with regard to functional recovery, neuronal survival, and autophagic flux. Compound C1 binds specifically to TFEB at its N-terminus and promotes its nuclear translocation without inhibiting mTOR activity. By activating TFEB, compound C1 enhances autophagy and lysosome biogenesis in vitro and in vivo [35].

In our analysis of locomotor function, SCI animals that were treated with compound C1 (SCI-CompC1) performed better starting 3 days after injury compared with the SCI-sal group (Fig. 4F) but similarly to the SCI-Guana group (Fig. 4F). Next, we determined the efficacy of compound C1 in promoting neuronal survival and TFEB nuclear translocation in RN neurons. We found that in SCI-CompC1 animals, the percentage of surviving neurons was higher than in SCI-sal (Fig. 4G) but insignificantly lower versus SCI-Guana (Fig. 4G). In a parallel qualitative and quantitative analysis of TFEB localization, the percentage of neurons with nuclear TFEB was significantly higher in SCI-CompC1 versus SCI-sal (Fig. 4H–I) and similar in SCI-Guana animals (Fig. 4H–I).

Notably, with regard to autophagy markers, LC3-II levels were similar between the SCI-CompC1, SCI-Guana, and SCI-sal groups (Fig. 4J), whereas p62/SQSTM1, which was upregulated in RN after SCI, decreased significantly compared to SCI-Sal (Fig. 4J) but similar to the levels observed in the SCI-Guana group (Fig. 4J). Moreover, both LAMP1 and LAMP2 levels rose significantly in SCI-CompC1 compared to SCI-Sal group (Fig. 4J) and, although LAMP1 was insignificantly higher than in SCI-Guana animals (Fig. 4J), LAMP2 levels were not significantly different (Fig. 4J).

Overall, these results suggest that TFEB is crucial in the regulation of autophagic flux in alleviating apoptotic cell death and promoting functional recovery after SCI.

## Discussion

The aim of this study was to examine the relationship between ER stress and autophagy and determine whether this crosstalk affects the course of secondary damage in remote regions and outcomes after SCI. Although several studies have evaluated the relationship between ER stress and autophagy in various experimental models of CNS injury [24, 25, 46, 47], their relationship in remote cell death after CNS injury has not been reported.

Using a hemisection model of SCI—a sensitive and reliable paradigm for evaluating supra-spinal changes after spinal injury—we found that ER stress and impaired autophagic flux are intertwined, contributing to remote apoptotic neuronal death. Further, we demonstrated that SCI-induced ER stress directly affects TFEB activity, which is responsible for the lysosomal impairment and blockade in autophagic flux. Enhancement of UPR by guanabenz also restores TFEB activity and, consequently, the autophagic machinery that protects remote neurons from death.

Following SCI, supra-spinal neurons experience a chronic injury state, and the relationship between atrophy and cell death has been largely debated over time generating contradictory results [6]. Counting method, tools to assess death, species, experimental model of SCI employed and age might account for these discrepancies. The cell loss that we observed in rubro-spinal neurons is based on unbiased stereology methods and by molecular analysis of key elements of the apoptotic cascade: cytocrome c release, caspase-3 activation. All of these indices confirmed that SCI-induced activation of the apoptotic cascade, as evidenced by caspase-3 activation and TUNEL assay, clearly indicating the commitment of rubro-spinal neurons irreversibly toward death that was significantly halted by guanabenz treatment.

Cell death due to the accumulation of misfolded proteins in the ER is observed in many pathological conditions, including CNS injury [24, 25, 48–50]. To cope with ER stress, damaged cells initiate the UPR, an adaptive signal transduction pathway that, depending on the level of damage, orchestrates the signals that are crucial for the survival or death of cells. Although ER stress is a common hallmark in secondary damage after SCI, whit a massive upregulation of key ER stress markers also rostral to the primary site of injury [51], our study is the first evidence that ER stress occurs even in regions that are remote but functionally connected to the primary injury site.

It is noteworthy that axonal damage activates several signaling pathways that transmit specific molecular messages from the site of injury to the soma of damaged neurons [52]. These mechanisms are essential in sensing the injury locally and for signaling such damage to the cell body to initiate the appropriate somatic responses. Although the mechanisms that induce ER stress in the soma of axotomized neurons are unknown, we can speculate that it can be activated indirectly or directly. In the first case, a warning fast retrograde transmissible signal can reach the soma, where it initiates the protein-folding stress response; alternatively, the ER stress might be induced locally in the axon and subsequently translocated to the cell body. However, we cannot exclude a combination of these two mechanisms, but the extension of this phenomenon and the mechanisms that contribute to the spread of signals near as well as far from the injury site need further investigations.

Autophagy is a highly conserved self-degradation pathway that is involved in the turnover of cytosolic constituents, long-lived proteins, and damaged organelles that are delivered to lysosomes for degradation [53]. Defective autophagy contributes to many diseases, including cancer, cardiovascular diseases, immune-mediated disorders, neurodegenerative diseases [54, 55], and TBI and SCI [56–61]. SCI elicits the robust accumulation of autophagosomes, as evidenced by increases in LC3 and p62 at the site of the injury [24, 25] and in distal regions [8].

In our study, we confirmed the accumulation of autophagosomes in RN neurons starting 1 day after injury and peaking at Day 5. Moreover, the accumulation of the substrate protein p62/SQSTM1, paralleling a decrease in the lysosomal proteins LAMP1 and LAMP2 confirmed that the accumulation of autophagosomes resulted from the inhibition of autophagic flux due to impaired lysosome biogenesis. Further, we demonstrated that the disruption in autophagy flux due to altered lysosome biogenesis paralleled the upregulation of ER stress markers. This observation extends previous findings on secondary damage [24, 25], suggesting a causal relationship between ER stress and disruptions in autophagic flux due to impaired lysosome biogenesis as demonstrated on various experimental disease models [27, 62, 63]. Since in this context the duration of ER stress appears to be an important factor in the transition from normal autophagic flux to its inhibition [26], we hypothesize that the sustained, prolonged, and unresolved ER stress/UPR failure induced by SCI causes the autophagy dysfunction. Although it was unknown whether the ER stress or disruption in autophagic flux occurred first in our study (because their kinetics overlap), our results clearly demonstrate that the relationship between these mechanisms likely potentiated the ensuing remote cell death after injury.

The link between ER stress and autophagy in response to SCI has not been clearly defined. In this context, most studies support the blockade of autophagic flux as the trigger of ER stress, causing cell death and leading to disease progression [24, 25]. Our results confirm a tight interaction between the two mechanisms and that ER stress and autophagy are intertwined, leading to a vicious cycle in the pathophysiology of SCI, as observed in other neurological pathologies [28–32]. This conclusion is supported by our pharmacological findings, in which the inhibition of a single factor—ER stress—by guanabenz, restored the autophagic flux and, consequently, improved remote neuronal survival and functional recovery. These changes were accompanied by increased lysosome biogenesis and autophagosome-lysosome fusion through the modulation of TFEB activity. Further, these findings confirm that ER stress/UPR and autophagy—although they are independent, vital cellular homeostatic mechanisms—share features and that altering the function of one of these systems can influence the other.

Guanabenz is a FDA-approved antihypertensive drug that can enhance UPR by keeping prolonged eIF2α phosphorylation and inhibition of protein synthesis in stressed cells [40], with neuroprotective effects in various CNS pathologies [33, 64, 65]. Notably, in our model, guanabenz treatment enhanced the UPR and improved autophagic flux. Although we hypothesize that it acts on autophagic flux indirectly, by enhancing UPR, we cannot exclude the possibility that guanabenz has efficacy against various signaling molecules and cells [66, 67], and, thus, also directly on autophagy.

Under stressful conditions, the MiT/TFE family, including MITF, TFEB, TFE3, and TFEC, regulates lysosome function and autophagy [41, 44, 45], the cellular responses to ER stress, and cell fate [68]. Notably, TFEB and TFE3 were recently identified as primary factors in the link between ER stress/UPR and autophagy [25, 69]. Our results extend previous findings and reveal that SCI-induced ER stress alters TFEB activity, but not TFE3, in remote neurons, which was restored by guanabenz through unknown mechanisms. TFEB activity is largely controlled by its subcellular localization, which is regulated primarily by phosphorylation [44, 70]. Our data show that after SCI, TFEB exists largely in the cytoplasm of axotomized neurons of the RN, suggesting that its activity is inhibited. This subcellular localization coincides with the upregulation of ER stress markers, the accumulation of autophagosomes due to lysosomal dysfunction, apoptotic remote cell death, and worse neurological recovery.

After SCI, chronic treatment with compound C1, a curcumin analog and a potent activator of TFEB through direct binding, promoted the nuclear translocation of TFEB and the degradation of the autophagy substrates p62/SQSTM1 and enhanced autophagy and lysosome biogenesis in axotomized neurons of the RN, as shown in other in vivo and in vitro models [35, 71]. Further, in our model, it improved neuronal survival and functional recovery, confirming the function of TFEB as a primary factor in linking the two mechanisms and rendering compound C1 a good neuroprotective drug candidate.

Establishing the link between the sparing of specific neuronal death in a given population and improvements in functional recovery after CNS lesions is challenging. Recovery after SCI requires rearrangements at many levels—in the spinal and supra-spinal regions—and it is not limited to a single brain structure or intracellular signaling pathways. After incomplete SCI, animals spontaneously recover locomotor function, and in our model, untreated SCI rats recovered progressively. In our study guanabenz treatment, as well as compound C1, in parallel with the delay in neurodegeneration, improves spontaneous recovery, based on our beam-walking data. However, we cannot exclude that the effects of guanabenz, and compound C1, might influence injury outcomes by acting on neural centers and cell populations that differ from those that we have considered. However, more work needs to be carried out in order to clarify this aspect.

In conclusion, our findings provide further evidence of the complexity of the mechanism of remote cell death and of the existence of functional interactions between ER stress/UPR, autophagy, and apoptotic remote cell death. Moreover, these findings implicate the recovery of ER proteostasis as a new target for future therapeutic interventions to act on multiple levels to ensure remote neuroprotection after SCI. Additional studies that target the links between ER stress and autophagy are needed to determine each of their contributions—particularly with regard to their kinetics—to the overall changes that are observed after injury to develop treatments for SCI. Furthermore, as it is noteworthy that the autophagic-lysosomal systems, both autophagy and chaperone-mediated-autophagy (CMA), and the UPR are functionally integrated for degrading damaged proteins to maintain cellular homeostasis [72, 73], we cannot exclude the involvement of CMA in our model. Indeed, considering that in several models of diseases [74, 75] CMA activity is upregulated as a compensatory response to autophagy failure, we cannot exclude that at later times after SCI the impaired autophagy can be counterbalanced by CMA. Further studies are needed to elucidate the possible interactions between autophagy, CMA and UPR and their respective roles in the mechanism of remote degeneration.

## Supplementary information

Supplentary figures

Checklist

## Data Availability

All data needed to evaluate the conclusions in the paper are present in the paper. Additional data related to this paper may be requested from the corresponding author.

## References

[1] Viscomi MT, Molinari M (2014). Remote neurodegeneration: multiple actors for one play. Mol Neurobiol.

[2] Carter AR, Patel KR, Astafiev SV, Snyder AZ, Rengachary J, Strube MJ (2012). Upstream dysfunction of somatomotor functional connectivity after corticospinal damage in stroke. Neurorehabil Neural Repair.

[3] Zhang J, Zhang Y, Xing S, Liang Z, Zeng J (2012). Secondary neurodegeneration in remote regions after focal cerebral infarction: a new target for stroke management?. Stroke.

[4] Kwon BK, Liu J, Messerer C, Kobayashi NR, McGraw J, Oschipok L (2002). Survival and regeneration of rubrospinal neurons 1 year after spinal cord injury. Proc Natl Acad Sci USA.

[5] Liu P-H, Wang Y-J, Tseng G-F (2003). Close axonal injury of rubrospinal neurons induced transient perineuronal astrocytic and microglial reaction that coincided with their massive degeneration. Exp Neurol.

[6] Hassannejad Z, Zadegan S, Shakouri-Motlagh A, Mokhatab M, Rezvan M, Sharif-Alhoseini M, et al. The fate of neurons after traumatic spinal cord injury in rats: a systematic review. Iran J Basic Med Sci 2018;21. 10.22038/ijbms.2018.24239.6052. 10.22038/IJBMS.2018.24239.6052 PMC6015255 29942443

[7] Latini L, Bisicchia E, Sasso V, Chiurchiù V, Cavallucci V, Molinari M (2014). Cannabinoid CB2 receptor (CB2R) stimulation delays rubrospinal mitochondrial-dependent degeneration and improves functional recovery after spinal cord hemisection by ERK1/2 inactivation. Cell Death Dis.

[8] Bisicchia E, Latini L, Cavallucci V, Sasso V, Nicolin V, Molinari M (2017). Autophagy inhibition favors survival of rubrospinal neurons after spinal cord hemisection. Mol Neurobiol.

[9] Seranova E, Connolly KJ, Zatyka M, Rosenstock TR, Barrett T, Tuxworth RI (2017). Dysregulation of autophagy as a common mechanism in lysosomal storage diseases. Essays Biochem.

[10] Malik BR, Maddison DC, Smith GA, Peters OM (2019). Autophagic and endo-lysosomal dysfunction in neurodegenerative disease. Mol Brain.

[11] Mizushima N, Levine B, Cuervo AM, Klionsky DJ (2008). Autophagy fights disease through cellular self-digestion. Nature.

[12] Kroemer G, Mariño G, Levine B (2010). Autophagy and the integrated stress response. Mol Cell.

[13] Brodsky JL, Skach WR (2011). Protein folding and quality control in the endoplasmic reticulum: recent lessons from yeast and mammalian cell systems. Curr Opin Cell Biol.

[14] Szegezdi E, Logue SE, Gorman AM, Samali A (2006). Mediators of endoplasmic reticulum stress‐induced apoptosis. EMBO Rep..

[15] Hetz C (2012). The unfolded protein response: controlling cell fate decisions under ER stress and beyond. Nat Rev Mol Cell Biol.

[16] Hayashi-Nishino M, Fujita N, Noda T, Yamaguchi A, Yoshimori T, Yamamoto A (2009). A subdomain of the endoplasmic reticulum forms a cradle for autophagosome formation. Nat Cell Biol.

[17] Ogata M, Hino S, Saito A, Morikawa K, Kondo S, Kanemoto S (2006). Autophagy is activated for cell survival after endoplasmic reticulum stress. Mol Cell Biol.

[18] Bernales S, McDonald KL, Walter P (2006). Autophagy counterbalances endoplasmic reticulum expansion during the unfolded protein response. PLoS Biol.

[19] Yorimitsu T, Nair U, Yang Z, Klionsky DJ (2006). Endoplasmic reticulum stress triggers autophagy. J Biol Chem.

[20] Yan F, Li J, Chen J, Hu Q, Gu C, Lin W (2014). Endoplasmic reticulum stress is associated with neuroprotection against apoptosis via autophagy activation in a rat model of subarachnoid hemorrhage. Neurosci Lett.

[21] Rashid H-O, Yadav RK, Kim H-R, Chae H-J (2015). ER stress: autophagy induction, inhibition and selection. Autophagy.

[22] Yorimitsu T, Klionsky DJ (2007). Endoplasmic reticulum stress: a new pathway to induce autophagy. Autophagy.

[23] Yan C, Liu J, Gao J, Sun Y, Zhang L, Song H (2020). Correction: IRE1 promotes neurodegeneration through autophagy-dependent neuron death in the Drosophila model of Parkinson’s disease. Cell Death Dis.

[24] Liu S, Sarkar C, Dinizo M, Faden AI, Koh EY, Lipinski MM (2015). Disrupted autophagy after spinal cord injury is associated with ER stress and neuronal cell death. Cell Death Dis.

[25] Zhou K, Zheng Z, Li Y, Han W, Zhang J, Mao Y (2020). TFE3, a potential therapeutic target for Spinal Cord Injury via augmenting autophagy flux and alleviating ER stress. Theranostics.

[26] González-Rodríguez Á, Mayoral R, Agra N, Valdecantos MP, Pardo V, Miquilena-Colina ME (2014). Impaired autophagic flux is associated with increased endoplasmic reticulum stress during the development of NAFLD. Cell Death Dis.

[27] Nakashima A, Cheng S-B, Kusabiraki T, Motomura K, Aoki A, Ushijima A (2019). Endoplasmic reticulum stress disrupts lysosomal homeostasis and induces blockade of autophagic flux in human trophoblasts. Sci Rep..

[28] Matus S, Lisbona F, Torres M, Leon C, Thielen P, Hetz C (2008). The stress rheostat: an interplay between the unfolded protein response (UPR) and autophagy in neurodegeneration. CMM.

[29] Cai Y, Arikkath J, Yang L, Guo M-L, Periyasamy P, Buch S (2016). Interplay of endoplasmic reticulum stress and autophagy in neurodegenerative disorders. Autophagy.

[30] Thangaraj A, Sil S, Tripathi A, Chivero ET, Periyasamy P, Buch S. Targeting endoplasmic reticulum stress and autophagy as therapeutic approaches for neurological diseases. Int Rev Cell Mol Biol. 2020;350:285–325. 10.1016/bs.ircmb.2019.11.001. 10.1016/bs.ircmb.2019.11.001 32138902

[31] Nakka VP, Prakash-babu P, Vemuganti R (2016). Crosstalk between endoplasmic reticulum stress, oxidative stress, and autophagy: potential therapeutic targets for acute CNS injuries. Mol Neurobiol.

[32] Yin Y, Sun G, Li E, Kiselyov K, Sun D (2017). ER stress and impaired autophagy flux in neuronal degeneration and brain injury. Ageing Res Rev.

[33] Wang L, Popko B, Tixier E, Roos RP (2014). Guanabenz, which enhances the unfolded protein response, ameliorates mutant SOD1-induced amyotrophic lateral sclerosis. Neurobiol Dis.

[34] Jiang H-Q, Ren M, Jiang H-Z, Wang J, Zhang J, Yin X (2014). Guanabenz delays the onset of disease symptoms, extends lifespan, improves motor performance and attenuates motor neuron loss in the SOD1 G93A mouse model of amyotrophic lateral sclerosis. Neuroscience.

[35] Song J-X, Sun Y-R, Peluso I, Zeng Y, Yu X, Lu J-H (2016). A novel curcumin analog binds to and activates TFEB in vitro and in vivo independent of MTOR inhibition. Autophagy.

[36] Viscomi MT, Florenzano F, Conversi D, Bernardi G, Molinari M (2004). Axotomy dependent purinergic and nitrergic co-expression. Neuroscience.

[37] Bordi M, Berg MJ, Mohan PS, Peterhoff CM, Alldred MJ, Che S (2016). Autophagy flux in CA1 neurons of Alzheimer hippocampus: increased induction overburdens failing lysosomes to propel neuritic dystrophy. Autophagy.

[38] La Barbera L, Vedele F, Nobili A, Krashia P, Spoleti E, Latagliata EC (2021). Nilotinib restores memory function by preventing dopaminergic neuron degeneration in a mouse model of Alzheimer’s Disease. Prog Neurobiol.

[39] Li H, Zhang X, Qi X, Zhu X, Cheng L (2019). Icariin inhibits endoplasmic reticulum stress-induced neuronal apoptosis after spinal cord injury through modulating the PI3K/AKT signaling pathway. Int J Biol Sci.

[40] Tsaytler P, Harding HP, Ron D, Bertolotti A (2011). Selective inhibition of a regulatory subunit of protein phosphatase 1 restores proteostasis. Science.

[41] Sardiello M, Palmieri M, di Ronza A, Medina DL, Valenza M, Gennarino VA (2009). A gene network regulating lysosomal biogenesis and function. Science.

[42] Yang M, Liu E, Tang L, Lei Y, Sun X, Hu J (2018). Emerging roles and regulation of MiT/TFE transcriptional factors. Cell Commun Signal.

[43] Bai L, Mei X, Wang Y, Yuan Y, Bi Y, Li G (2017). The role of netrin-1 in Improving functional recovery through autophagy stimulation following spinal cord injury in rats. Front Cell Neurosci.

[44] Settembre C, Di Malta C, Polito VA, Arencibia MG, Vetrini F, Erdin S (2011). TFEB links autophagy to lysosomal biogenesis. Science.

[45] Settembre C, Zoncu R, Medina DL, Vetrini F, Erdin S, Erdin S (2012). A lysosome-to-nucleus signalling mechanism senses and regulates the lysosome via mTOR and TFEB: self-regulation of the lysosome via mTOR and TFEB. EMBO J.

[46] Gao B, Zhang X, Han R, Zhang T, Chen C, Qin Z (2013). The endoplasmic reticulum stress inhibitor salubrinal inhibits the activation of autophagy and neuroprotection induced by brain ischemic preconditioning. Acta Pharm Sin.

[47] Wang D-Y, Hong M-Y, Pei J, Gao Y-H, Zheng Y, Xu X (2021). ER stress mediated‑autophagy contributes to neurological dysfunction in traumatic brain injury via the ATF6 UPR signaling pathway. Mol Med Rep..

[48] Aufenberg C, Wenkel S, Mautes A, Paschen W (2005). Short communication: spinal cord trauma activates processing of xbp1 mRNA indicative of endoplasmic reticulum dysfunction. J Neurotrauma.

[49] Ohri SS, Maddie MA, Zhang Y, Shields CB, Hetman M, Whittemore SR (2012). Deletion of the pro-apoptotic endoplasmic reticulum stress response effector CHOP does not result in improved locomotor function after severe contusive spinal cord injury. J Neurotrauma.

[50] Ohri SS, Hetman M, Whittemore SR (2013). Restoring endoplasmic reticulum homeostasis improves functional recovery after spinal cord injury. Neurobiol Dis.

[51] Valenzuela V, Collyer E, Armentano D, Parsons GB, Court FA, Hetz C (2012). Activation of the unfolded protein response enhances motor recovery after spinal cord injury. Cell Death Dis.

[52] Abe N, Cavalli V (2008). Nerve injury signaling. Curr Opin Neurobiol.

[53] Mizushima N (2007). Autophagy: process and function. Genes Dev.

[54] Levine B, Kroemer G (2019). Biological functions of autophagy genes: a disease perspective. Cell.

[55] Zhang J (2013). Autophagy and mitophagy in cellular damage control. Redox Biol.

[56] Smith CM, Chen Y, Sullivan ML, Kochanek PM, Clark RSB (2011). Autophagy in acute brain injury: feast, famine, or folly?. Neurobiol Dis.

[57] Lipinski M, Wu J (2015). Modification of autophagy-lysosomal pathway as a neuroprotective treatment for spinal cord injury. Neural Regen Res.

[58] Galluzzi L, Bravo-San Pedro JM, Blomgren K, Kroemer G (2016). Autophagy in acute brain injury. Nat Rev Neurosci.

[59] Wolf MS, Bayır H, Kochanek PM, Clark RSB (2019). The role of autophagy in acute brain injury: a state of flux?. Neurobiol Dis.

[60] Wu J, Lipinski MM (2019). Autophagy in neurotrauma: good, bad, or dysregulated. Cells.

[61] Lipinski MM, Wu J, Faden AI, Sarkar C (2015). Function and mechanisms of autophagy in brain and spinal cord trauma. Antioxid Redox Signal.

[62] Miyagawa K, Oe S, Honma Y, Izumi H, Baba R, Harada M (2016). Lipid-induced endoplasmic reticulum stress impairs selective autophagy at the step of autophagosome-lysosome fusion in hepatocytes. Am J Pathol.

[63] Fang C, Weng T, Hu S, Yuan Z, Xiong H, Huang B (2021). IFN-γ-induced ER stress impairs autophagy and triggers apoptosis in lung cancer cells. OncoImmunology.

[64] Dash PK, Hylin MJ, Hood KN, Orsi SA, Zhao J, Redell JB (2015). Inhibition of eukaryotic initiation factor 2 alpha phosphatase reduces tissue damage and improves learning and memory after experimental traumatic brain injury. J Neurotrauma.

[65] Sun X, Aimé P, Dai D, Ramalingam N, Crary JF, Burke RE (2018). Guanabenz promotes neuronal survival via enhancement of ATF4 and parkin expression in models of Parkinson disease. Exp Neurol.

[66] Thompson KK, Tsirka SE (2020). Guanabenz modulates microglia and macrophages during demyelination. Sci Rep..

[67] Takigawa S, Chen A, Nishimura A, Liu S, Li B-Y, Sudo A (2016). Guanabenz downregulates inflammatory responses via eIF2α dependent and independent signaling. IJMS.

[68] Martina JA, Diab HI, Li H, Puertollano R (2014). Novel roles for the MiTF/TFE family of transcription factors in organelle biogenesis, nutrient sensing, and energy homeostasis. Cell Mol Life Sci.

[69] Zhang Z, Qian Q, Li M, Shao F, Ding W-X, Lira VA (2021). The unfolded protein response regulates hepatic autophagy by sXBP1-mediated activation of TFEB. Autophagy.

[70] Puertollano R, Ferguson SM, Brugarolas J, Ballabio A. The complex relationship between TFEB transcription factor phosphorylation and subcellular localization. EMBO J. 2018;37. 10.15252/embj.201798804. 10.15252/embj.201798804 PMC5983138 29764979

[71] Zhuang X-X, Wang S-F, Tan Y, Song J-X, Zhu Z, Wang Z-Y (2020). Pharmacological enhancement of TFEB-mediated autophagy alleviated neuronal death in oxidative stress-induced Parkinson’s disease models. Cell Death Dis.

[72] Schreiber A, Peter M (2014). Substrate recognition in selective autophagy and the ubiquitin–proteasome system. Biochimica et Biophysica Acta (BBA) - Mol Cell Res.

[73] Nedelsky NB, Todd PK, Taylor JP (2008). Autophagy and the ubiquitin-proteasome system: collaborators in neuroprotection. Biochimica et Biophysica Acta (BBA) - Mol Basis Dis.

[74] Li W, Zhu J, Dou J, She H, Tao K, Xu H (2017). Phosphorylation of LAMP2A by p38 MAPK couples ER stress to chaperone-mediated autophagy. Nat Commun.

[75] Li W, Yang Q, Mao Z. Signaling and induction of chaperone-mediated autophagy by the endoplasmic reticulum under stress conditions. Autophagy. 2018:1–3. 10.1080/15548627.2018.1444314. 10.1080/15548627.2018.1444314 PMC6103395 29771174

