# Supplementary material for: Restoration of ER proteostasis attenuates remote apoptotic cell death after spinal cord injury by reducing autophagosome overload
Source: Cell Death Dis. 2022 Apr 20;13(4):381. doi: 10.1038/s41419-022-04830-9 (PMC9021197; doi:10.1038/s41419-022-04830-9)
Supplement: Supplementary file 1 — Supplentary figures [file 41419_2022_4830_MOESM1_ESM.docx]

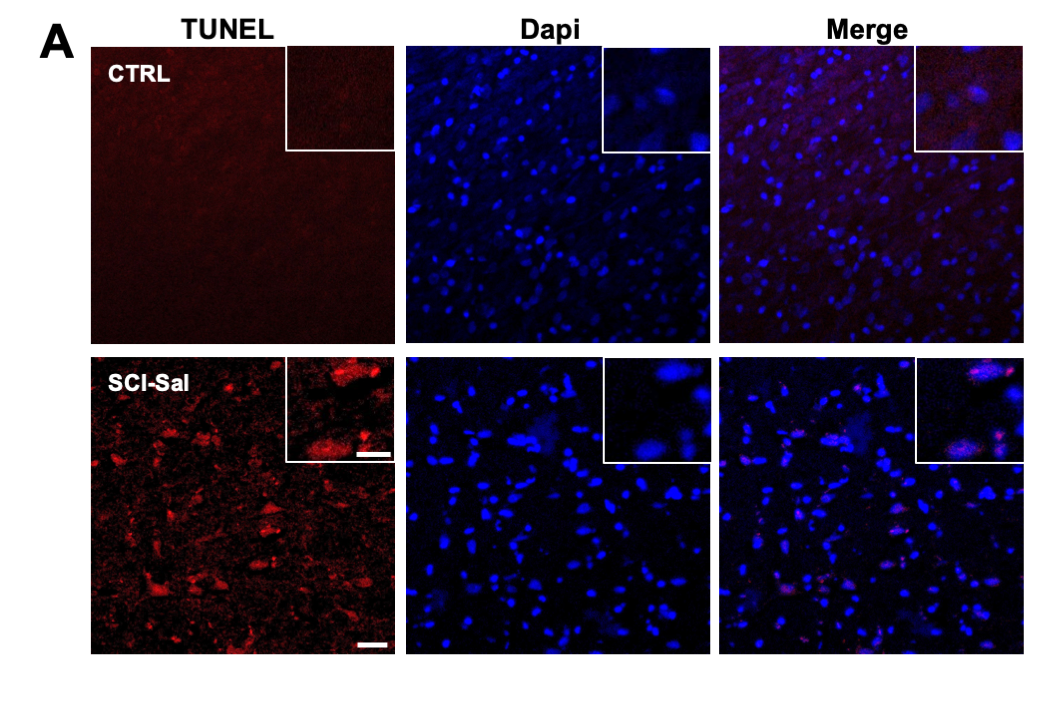


Supplementary Fig. 1

**Supplementary Fig. 1**. Representative confocal images of TUNEL staining (red) and Dapi- counterstaining (blue) from RN of CTRL and SCI-Sal animals showing the absence of apoptotic nuclei in CTRL and the presence in SCI-Sal animals. (scale bar=20μm; insets: scale bar=10μm).


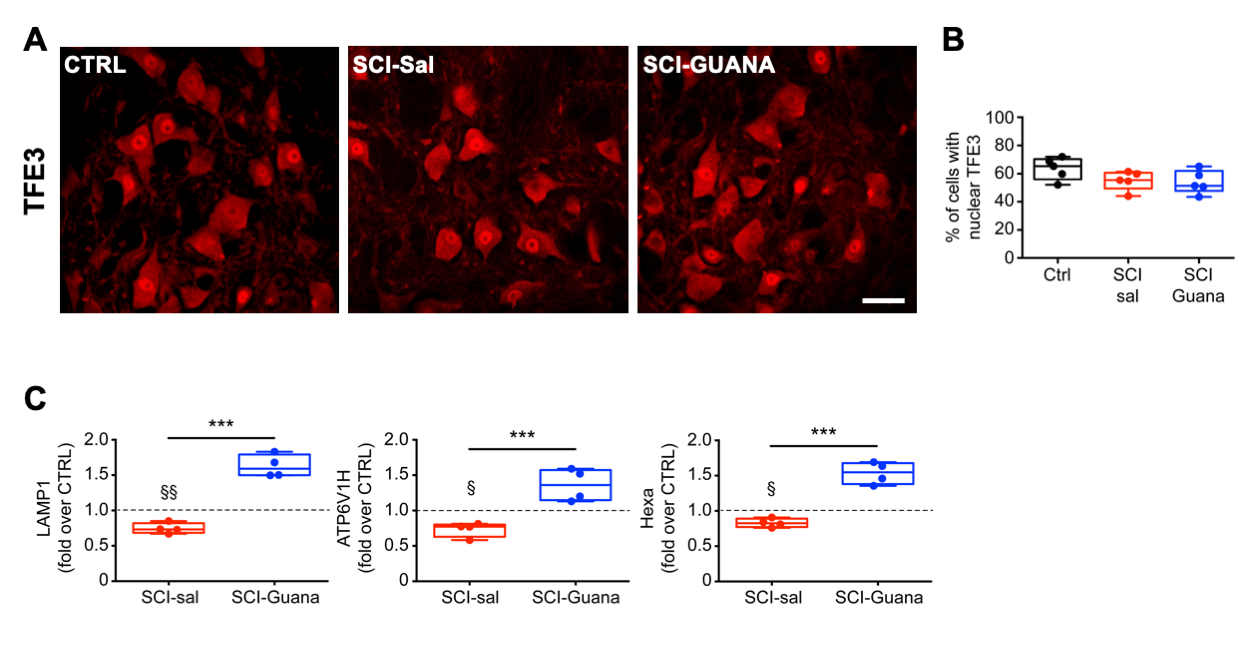


Supplementary Fig. 2

**Supplementary Fig. 2**. (**A**) Representative confocal images of TFE3 immunofluorescence from RN showing the compartmentalization of TFE3 immunostaining in neurons of CTRL, SCI-sal, and SCI-Guana groups (scale bar=20μm). (**B**) Box and whisker plots showing the percentage of neurons of RN with nuclear expression of TFE3 in CTRL, SCI-sal, and SCI-Guana (n=5 sections/rat; N=5 rats/group; m/f, = 3/2; One-way ANOVA p=0.7269). (**C**) Box-and-whisker plots of Lamp1, Lamp2, ATP6V1H, and Hexa mRNA level in SCI-sal and SCI-Guana expressed as fold over CTRL (N = 4 rats per group; One-way ANOVA, Lamp1 p<0.001; Lamp2 p<0.01; ATP6V1H p<0.01; Hexa p<0.001) *** p < 0.001; ** p< 0.01
